# Novel disease-modifying anti-rheumatic drug iguratimod suppresses chronic experimental autoimmune encephalomyelitis by down-regulating activation of macrophages/microglia through an NF-κB pathway

**DOI:** 10.1038/s41598-018-20390-5

**Published:** 2018-01-31

**Authors:** Guangrui Li, Ryo Yamasaki, Mei Fang, Katsuhisa Masaki, Hirofumi Ochi, Takuya Matsushita, Jun-ichi Kira

**Affiliations:** 1 Department of Neurology, Neurological Institute, Graduate School of Medical Sciences, Kyushu University, Fukuoka, 812-8582 Japan; 2 Department of Geriatric Medicine and Neurology, Ehime University Graduate School of Medicine, Matsuyama, 791-0295 Japan

## Abstract

We aimed to elucidate the effects of iguratimod, a widely used anti-rheumatic drug with no severe side effects, on chronic experimental autoimmune encephalomyelitis (EAE), an animal model of multiple sclerosis (MS). Iguratimod was orally administered to mice immunised with myelin oligodendrocyte glycoprotein peptide 35–55. Preventive administration of iguratimod from the time of immunisation was found to markedly reduce the clinical severity of acute and chronic EAE. Pathologically, iguratimod treatment significantly reduced demyelination and infiltration of CD3^+^ T, F4/80^+^, and CD169^+^ cells into the spinal cord, and suppressed macrophage/microglia activation in the parenchyma at the acute and chronic stages compared with vehicle treatment. Therapeutic administration of iguratimod after the onset of clinical symptoms significantly ameliorated the clinical severity of chronic EAE and reduced demyelination, T helper (Th)1/Th17 cell infiltration, macrophage/microglia activation, and nuclear factor (NF)-κB p65 and cyclooxygenase-2 expression in the spinal cord. *In vitro*, iguratimod treatment inhibited nuclear translocation of NF-κB p65 and down-regulated pro-inflammatory responses in macrophages and microglia. Our results suggest that iguratimod ameliorates acute and chronic EAE by suppressing inflammatory cell infiltration and immune cell activation, partly through inhibition of NF-κB p65, supporting the therapeutic potential of this drug for not only acute, but also chronic MS.

## Introduction

Multiple sclerosis (MS) is the most common inflammatory demyelinating disease of the central nervous system (CNS) and is characterised by recurrent episodes of focal demyelinating symptoms^1,2^. It usually begins as relapsing-remitting MS (RRMS), and then progresses after a few decades to secondary progressive MS (SPMS) characterised by irreversible accumulation of neurological deficits^1,2^. MS is recognised as a disease mediated by the adaptive immune system, wherein T helper (Th) 1 and Th17 cells propagate immune responses against self-antigens in the CNS that mediate the pathological process in RRMS^3–6^. Consequently, recent disease-modifying drugs (DMDs) that target the adaptive immune system, such as fingolimod^7–10^, dimethyl fumarate^11,12^, natalizumab^13^, and anti-CD20 monoclonal antibodies^14,15^, are highly effective for RRMS and widely used in clinical practice. However, these DMDs exhibit very limited efficacy against SPMS^16,17^, and occasionally cause the fatal complication progressive multifocal leukoencephalopathy (PML)^18^. Thus, DMDs that are safe and beneficial against both RRMS and SPMS are desired. In particular, oral DMDs are preferable because the progressive phase begins to develop at a very early stage when patients suffer from minimal disability^19–21^.

The discrepant effects of DMDs between RRMS and SPMS suggests that other immune mechanisms are involved in the progressive form of MS. Indeed, several lines of evidence indicate sustained activation of macrophages and microglia within the CNS compartment of progressive MS^20,22^, both of which are important effector cells for neurodegeneration as well as demyelination^21^. These findings suggest that down-regulation of activated macrophages/microglia may be a new therapeutic strategy for treatment of chronic progressive MS.

Iguratimod was originally reported to be a selective inhibitor of cyclooxygenase (COX)-2 that inhibits the synthesis of pro-inflammatory prostaglandins (PGs)^23–25^. It is currently in clinical use for treatment of rheumatoid arthritis as an oral small-molecule anti-rheumatic drug, and has been administered to around 100,000 patients in Japan and China with no occurrence of PML or severe adverse effects. In addition to its anti-inflammatory properties arising from the COX-2 inhibitory activity, iguratimod also acts as an immunomodulatory agent. Previous studies revealed that iguratimod suppresses the production of pro-inflammatory cytokines, such as interleukin (IL)-1, IL-6, IL-8, and tumour necrosis factor (TNF)-α, from activated monocytes/macrophages *in vitro*^26,27^. Through these anti-inflammatory and immunomodulatory functions, preventive administration of iguratimod was shown to decrease the severity of acute experimental autoimmune encephalomyelitis (EAE) by inhibiting proliferation of autoreactive T cells and production of pro-inflammatory cytokines from peripheral immune cells^28^. However, it remains to be elucidated whether preventive or therapeutic administration of iguratimod is effective for chronic EAE, which is regarded as a more appropriate model for SPMS.

Therefore, in the present study, we investigated the effects of iguratimod on mice with chronic EAE induced by immunisation with a peptide comprising amino acids 35–55 of myelin oligodendrocyte glycoprotein (MOG_35–55_)^29–32^. We found that either preventive or therapeutic administration of iguratimod ameliorated the severity of EAE in both the acute and chronic phases, associated with decreased inflammatory cell infiltration into the spinal cord and suppression of macrophage/microglial activation through a nuclear factor (NF)-κB pathway. These findings suggest the therapeutic potential of oral iguratimod for not only acute, but also chronic MS.

## Results

### Preventive administration of iguratimod initiated at the day of immunisation ameliorates acute and chronic EAE by suppressing inflammatory cell infiltration and microglial activation

**Figure 1:**
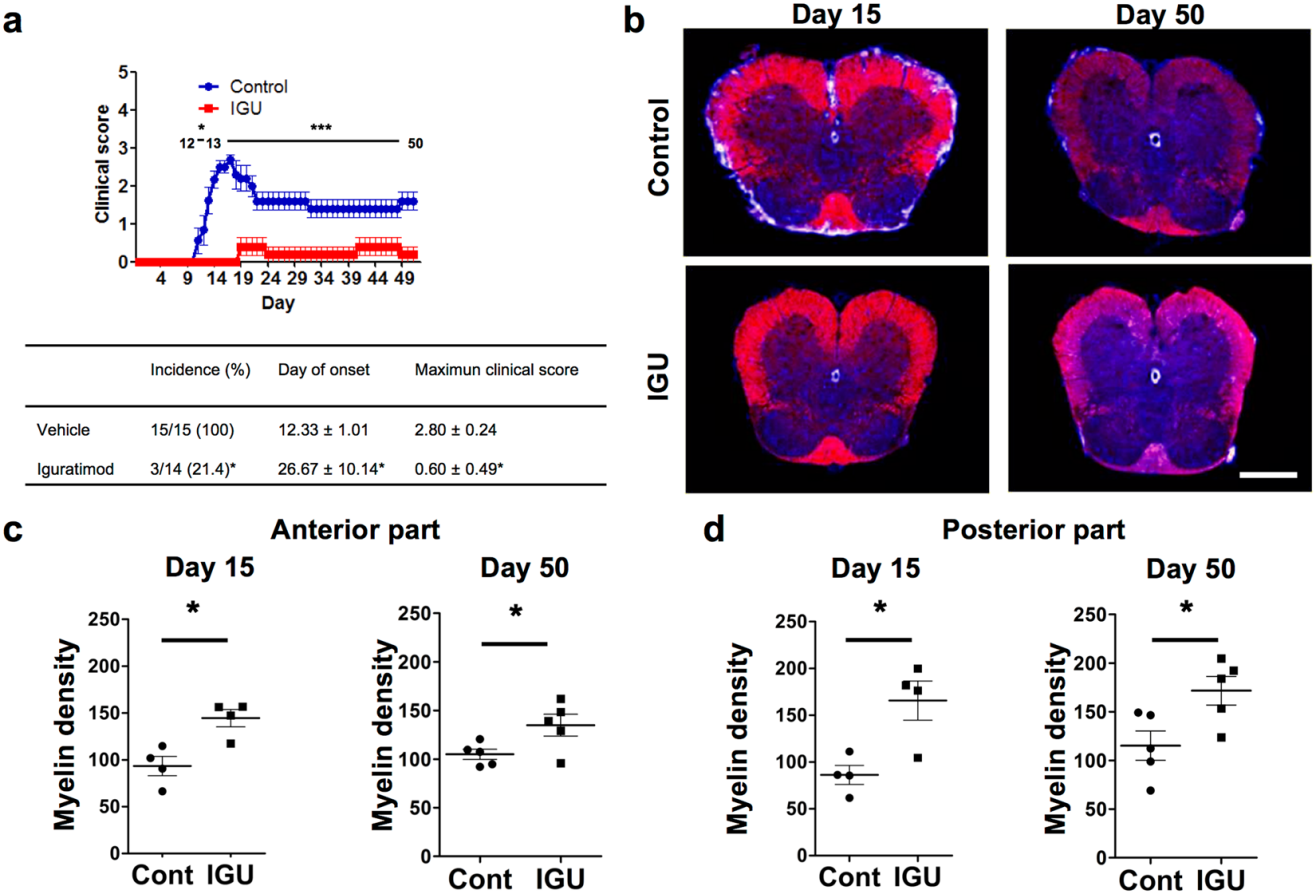
Iguratimod treatment prevents the development of MOG_35–55_-induced EAE in C57BL/6 mice and inhibits its related demyelination. Oral iguratimod was administered daily started from the day of immunisation. (**a**) EAE clinical scores (means ± SEM) over time in mice treated with iguratimod (filled squares, *n* = 14) or CMC vehicle (filled circles, *n* = 15). Horizontal bars in the graph show the periods of days with significant differences between the two groups. The lower table shows the following parameters as means ± SEM: incidence, day of onset, and maximum clinical score of each group. *p < 0.05, CMC vehicle-treated mice versus iguratimod-treated mice. (**b**) Cryostat cross-sections (20-µm thickness) of the lumbar spinal cord of iguratimod-treated mice and vehicle-treated mice. DAPI is shown in blue and myelin staining is shown in red. Scale bar: 500 µm. (**c**) Quantification of myelin density in the anterior white matter at day 15 (*n* = 4 per group) and day 50 (*n* = 5 per group) (identified by myelin staining). (**d**) Quantification of myelin density in posterior parts of the white matter at day 15 (*n* = 4 per group) and day 50 (*n* = 5 per group) (identified by myelin staining).

To investigate the effect of iguratimod on the development of EAE, iguratimod at 30 mg/kg body weight was orally administered daily to C57BL/6 mice immunised with MOG_35–55_ from the day of immunisation to the end of the experiment. Oral administration of iguratimod significantly delayed the onset of disease and reduced the incidence of EAE (Fig. 1a). Moreover, iguratimod markedly suppressed the disease severity assessed by the EAE clinical scores during the treatment period (*p* < 0.0001; Fig. 1a). Histopathological examinations performed at early (day 15) and late (day 50) time points revealed that the degree of demyelination, evaluated by myelin staining, was significantly reduced in iguratimod-treated mice compared with control carboxymethylcellulose (CMC) vehicle-treated mice at both time points (Fig. 1b–d).

**Figure 2:**
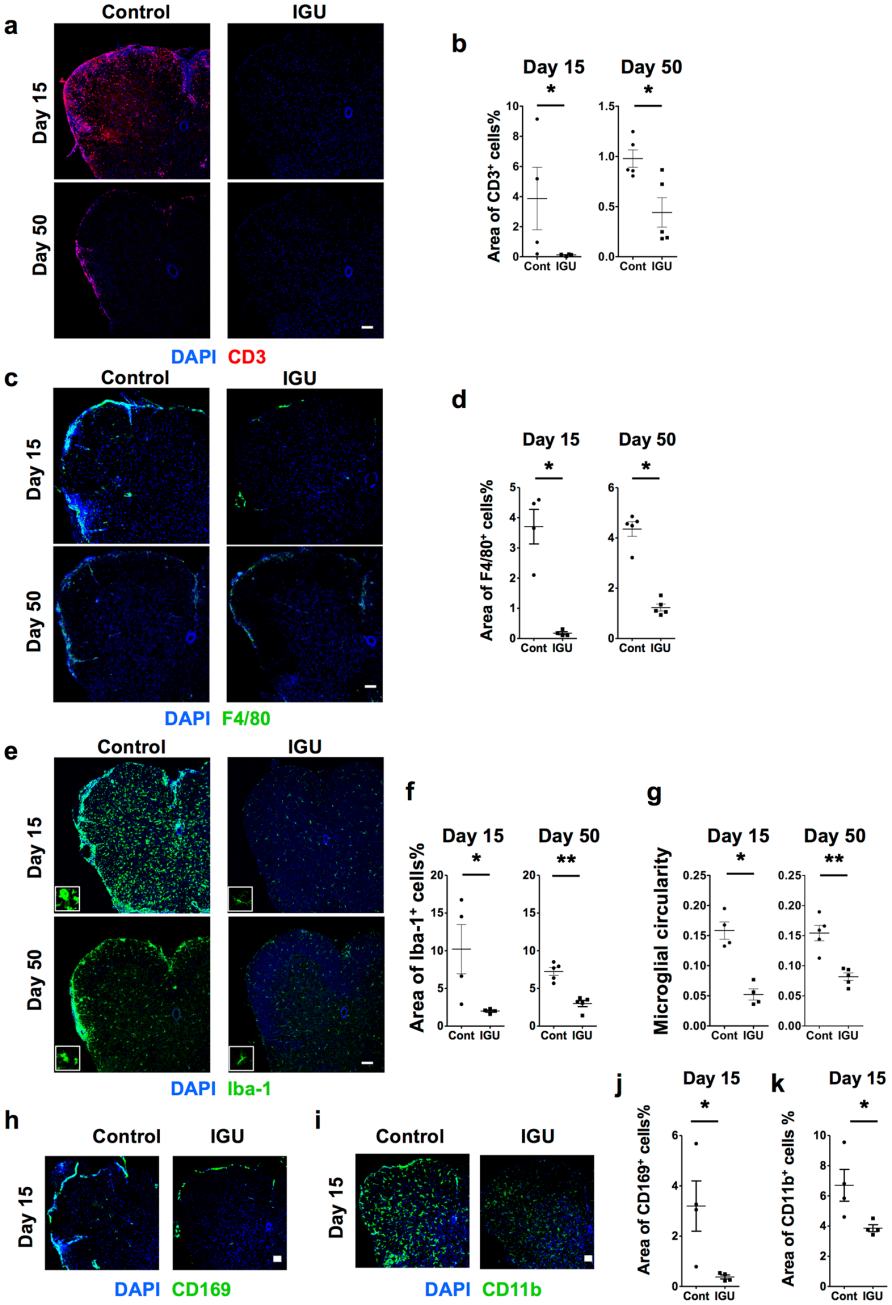
Preventive iguratimod administration suppresses inflammatory cell infiltration and microglial activation in MOG_35–55_-induced EAE. (**a**) Iguratimod treatment suppresses DAPI^+^CD3^+^ cell infiltration in the lumbar spinal cord of EAE mice. Scale bar: 100 μm. (**b**) Areas of DAPI^+^CD3^+^ cells quantified at day 15 (n = 4 per group) and day 50 (n = 5 per group). (**c**) Iguratimod treatment suppresses DAPI^+^F4/80^+^ cell infiltration in the lumbar spinal cord of EAE mice. Scale bar: 100 μm. (**d**) Areas of DAPI^+^F4/80^+^ cells quantified at day 15 (n = 4 per group) and day 50 (n = 5 per group). (**e**) Iguratimod treatment suppresses Iba-1 up-regulation in the lumbar spinal cord of mice with EAE. Scale bar: 100 μm. (**f**) Areas of DAPI^+^Iba-1^+^ cells quantified at day 15 (n = 4 per group) and day 50 (n = 5 per group). (**g**) Microglial cell circularity quantified at day 15 (n = 4 per group) and day 50 (n = 5 per group). (**h**) Iguratimod treatment suppresses DAPI^+^CD169^+^ cell infiltration in the lumbar spinal cord of EAE mice. Scale bar: 100 μm. (**i**) Iguratimod treatment suppresses DAPI^+^CD11b^+^ cell infiltration in the lumbar spinal cord of EAE mice. Scale bar: 100 μm. (**j**) Areas of DAPI^+^CD169^+^ cells quantified at day 15 (n = 4 per group). (**k**) Areas of CD11b^+^ cells quantified at day 15 (n = 4 per group). *p < 0.05, **p < 0.01. Error bars indicate SEM. An unpaired Student’s *t*-test was used for analysis.

Next, inflammatory cell infiltration into the spinal cord was analysed by immunofluorescence staining. At the early time point, infiltration of T cells (CD3^+^; Fig. 2a,b) and macrophages (F4/80^+^ or CD169^+^; Fig. 2c,d,h, and j) into the spinal cord of iguratimod- treated mice was almost completely abolished, and there were few Iba-1^+^ microglia in the parenchyma (Fig. 2e,f). We also found that CD11b^+^ cells, including both of macrophages and microglia, were significantly decreased in iguratimod-treated mice compared with vehicle-treated mice (Fig. 2I,k, *p* = 0.00386). At the late time point, although accumulation of leukocytes was observed in the spinal cord of iguratimod-treated mice, cellular infiltration was less pronounced compared with vehicle-treated mice (Fig. 2a–d). As a result, the number of Iba-1^+^ microglia in the spinal cord was significantly decreased in iguratimod-treated mice compared with vehicle-treated mice (Fig. 2e,f). In vehicle-treated mice, Iba-1^+^ microglia were characterized by enlarged cell bodies, a few short processes, if any, and often several filopodia at the early time point (day 15; Fig. 2e, inset), indicating that the microglia were in the activated state (amoeboid or bushy phenotype). Meanwhile, Iba-1^+^ microglia in iguratimod-treated mice had thin soma and delicate radially-projecting processes (Fig. 2e, inset), indicating that the microglia were in the resting state (ramified phenotype)^33^. Correspondingly, Iba-1^+^ microglia in iguratimod-treated mice showed lower circularity scores than those in vehicle-treated mice (*p* = 0.0286 at day 15; Fig. 2g). At the late time point (day 50), microglia in vehicle-treated mice were hypertrophic with thick short processes (Fig. 2e, inset), indicating that the microglia remained in the activated state. By contrast, few activated microglia were detected in iguratimod-treated mice, and the majority of the microglia were still in the ramified inactivated phenotype (Fig. 2e, inset). These Iba-1^+^ microglia in iguratimod-treated mice also showed lower circularity scores than those in vehicle-treated mice (*p* = 0.0079 at day 50; Fig. 2g).

### Therapeutic administration of iguratimod suppresses the chronic phase of EAE by inhibiting leukocyte infiltration and microglia activation

**Figure 3:**
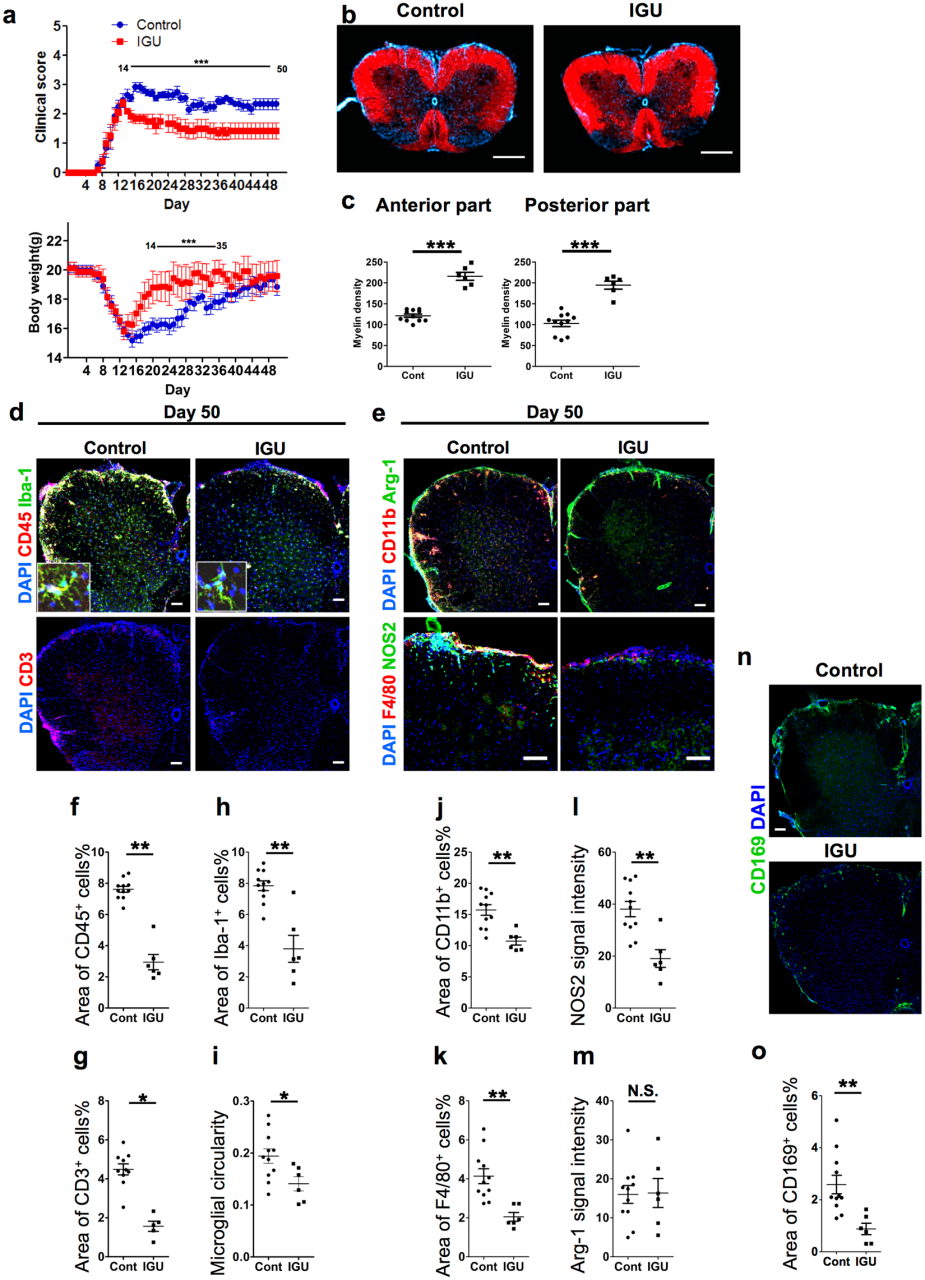
Therapeutic administration of iguratimod improves the severity of chronic EAE by suppressing inflammatory cell infiltration and microglia activation. Oral iguratimod was administered daily starting on the day of clinical onset (10 days after immunisation). (**a**) EAE clinical scores (means ± SEM) and body weights (means ± SEM) over time in mice treated with iguratimod (filled squares, *n* = 6) or CMC vehicle (filled circles, *n* = 11). Horizontal bars in the graphs show the periods of days with significant differences between the two groups. (**b**) Cryostat cross-sections (20 µm thickness) of the lumbar spinal cord of iguratimod-treated mice and vehicle-treated mice. DAPI is shown in blue and myelin staining is shown in red. Scale bar: 500 µm. (**c**) Quantification of myelin density identified by myelin staining in the anterior or posterior parts of the white matter at day 50 (*n* = 11 for control group; *n* = 6 for IGU group). (**d**) Iguratimod treatment after the onset of EAE suppresses DAPI^+^CD45^+^ and DAPI^+^CD3^+^ cell infiltration and decreases Iba-1 fluorescence in the lumbar spinal cord of mice with chronic EAE at day 50. Scale bar: 100 µm. (**e**) Iguratimod treatment after the onset of EAE decreases DAPI^+^F4/80^+^ and DAPI^+^CD11b^+^ cells and NOS2 fluorescence, but does not change arginase-1 fluorescence in the lumbar spinal cord of mice with chronic EAE at day 50. Scale bar: 100 µm. (**f**) Areas of CD45^+^ cells quantified at day 50 (*n* = 11 for control group, *n* = 6 for IGU group). (**g**) Areas of CD3^+^ cells quantified at day 50 (*n* = 11 for control group; *n* = 6 for IGU group). (**h**) Areas of Iba-1^+^ cells quantified at day 50 (*n* = 11 for control group; *n* = 6 for IGU group). (**i**) Microglial cell circularity (*n* = 11 for control group; *n* = 6 for IGU group). (**j**) Areas of CD11b^+^ cells quantified at day 50 (*n* = 11 for control group; *n* = 6 for IGU group). (**k**) Areas of F4/80^+^ cells quantified at day 50 (*n* = 11 for control group; *n* = 6 for IGU group). (**l**) NOS2 signal intensity quantified at day 50 (*n* = 11 for control group; *n* = 6 for IGU group). (**m**) Arginase-1 signal intensity quantified at day 50 (*n* = 11 for control group; *n* = 6 for IGU group). (**n**) Iguratimod treatment after the onset of EAE suppresses DAPI^+^/CD169^+^ cell infiltration in the lumbar spinal cord of mice with chronic EAE at day 50. (**o**) Areas of CD169^+^ cells quantified at day 5 (*n* = 11 for control group; *n* = 6 for IGU group). *p < 0.05, **p < 0.01, ***p < 0.0001. Error bars indicate SEM. An unpaired Student’s *t*-test was used for analyses.

In this model, the peak of acute neurological symptoms was followed by a stable chronic phase of the disease. The acute phase was characterised by severe disease activity with significant body weight loss, and the chronic phase was characterised by moderate disease severity with body weight recovery. To investigate the effects of iguratimod on chronic EAE, iguratimod was administered orally at 50 mg/kg body weight twice a day from the day of clinical onset (day 10) to the end of the experiment. Iguratimod treatment initiated after the symptom onset significantly attenuated the disease severity from day 15 compared with vehicle-treated mice (*p* < 0.0001; Fig. 3a, upper). Iguratimod-treated mice had significantly lower mean clinical EAE scores compared with vehicle-treated mice (1.75 ± 0.56 versus 2.73 ± 0.39 at day 20; 1.42 ± 0.61 versus 2.25 ± 0.51 at day 35; 1.42 ± 0.61 versus 2.33 ± 0.53 at day 50). In addition, body weight loss recovered more quickly in iguratimod-treated mice than in vehicle-treated mice (Fig. 3a, lower).

Histopathological examinations of the spinal cord were performed at day 50. Immunofluorescence staining of lumbar sections revealed significantly less demyelination in both the anterior and posterior parts of the lumbar spinal cord in iguratimod-treated mice compared with vehicle-treated mice (*p* < 0.0001 and *p* < 0.0001, respectively; Fig. 3b,c). Accumulation of leukocytes (CD45^+^) including T cells (CD3^+^) and macrophages (F4/80^+^ or CD169^+^) within the spinal cord was significantly decreased in iguratimod-treated mice compared with vehicle-treated mice (*p* = 0.0134 for CD45^+^ leukocytes, Fig. 3d,f; *p* = 0.0352 for CD3^+^ T cells, Fig. 3d,g; *p* = 0.0017 for F4/80^+^ macrophages, Fig. 3e,k; *p* = 0.0046 for CD169^+^ macrophages, Fig. 3n,o). We also found that CD11b^+^ cells, including both macrophages and microglia, were significantly decreased in iguratimod-treated mice compared with vehicle-treated mice (*p* = 0.0012, Fig. 3e,j). The number of microglia determined by Iba-1 immunostaining in the spinal cord was significantly decreased in iguratimod-treated mice compared with vehicle-treated mice (*p* = 0.0002, Fig. 3d,h). At the end of the treatment (day 50), Iba-1^+^ microglia in vehicle-treated mice showed a hypertrophic morphology with thick short processes (Fig. 3d, inset), indicating that the microglia remained in the activated state. By contrast, the majority of microglia in iguratimod-treated mice had decreased in size and exhibited a non-hypertrophic (inactivated) morphology (Fig. 3d, inset). Correspondingly, Iba-1^+^ microglia in iguratimod-treated mice showed lower circularity scores than those in vehicle-treated mice (*p* = 0.0394, Fig. 3i). We also found that the M1 marker inducible nitric oxide synthase (NOS2), which is critical for inflammation, was significantly decreased in the spinal cord of iguratimod-treated mice compared with vehicle-treated mice, while the M2 marker arginase-1 was unaffected (*p* = 0.0011 for NOS2, *p* = 0.9295 for arginase-1, Fig. 3e,l,m). These findings indicate that iguratimod initiated after the clinical onset can attenuate the disease severity of chronic EAE through the down-regulation of inflammatory infiltration and microglial activation.

### Iguratimod decreases COX-2 and NF-κB p65 in the chronic EAE spinal cord

**Figure 4:**
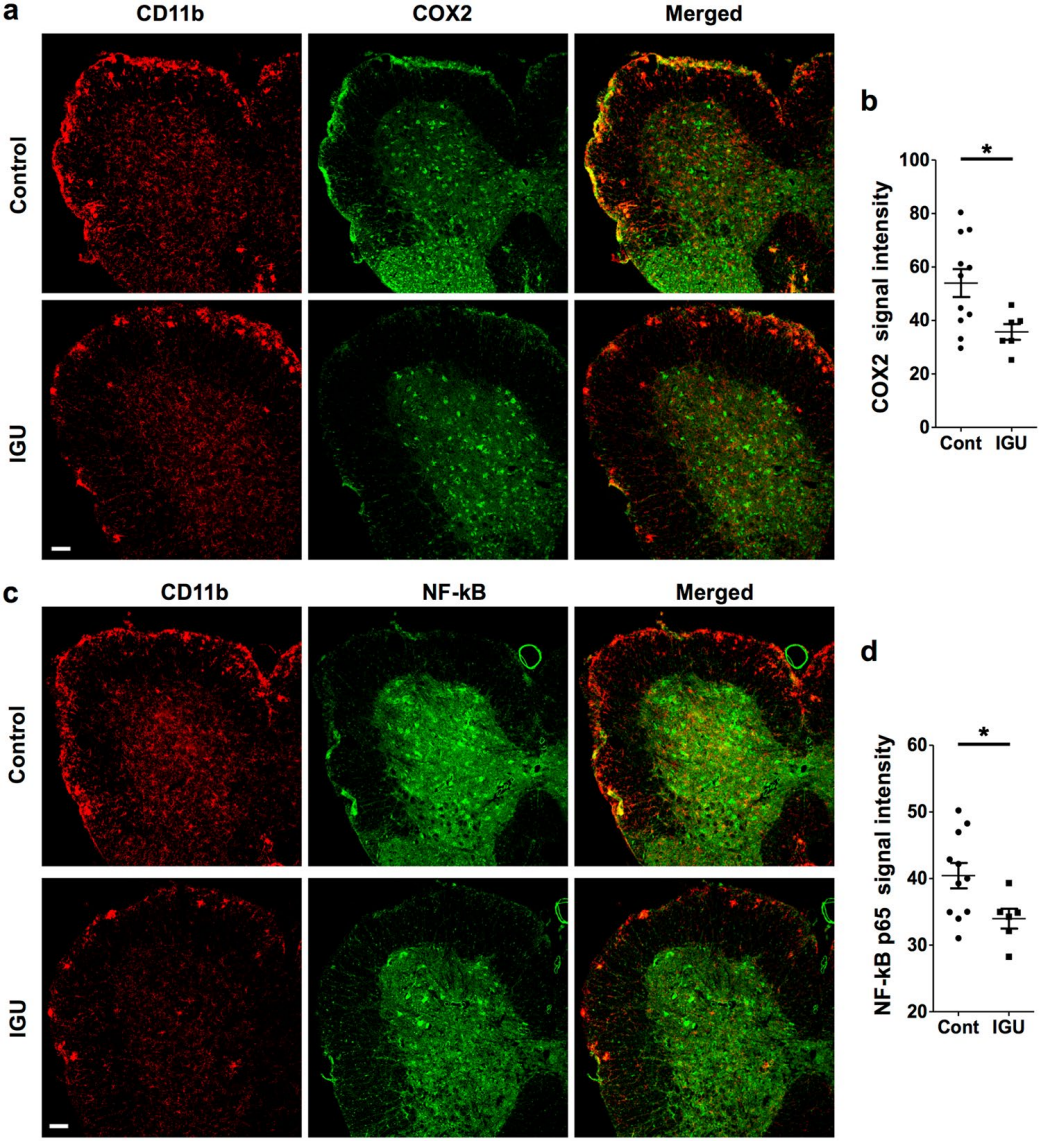
Therapeutic administration of iguratimod suppresses COX-2 and NF-κB p65 expression in the EAE spinal cord. (**a**) Iguratimod treatment after the onset of EAE decreases COX-2 fluorescence intensity. (**b**) COX-2 signal intensity was quantified at day 50 (*n* = 11 for control group; *n* = 6 for IGU group). (**c**) Iguratimod treatment after the onset of EAE decreases NF-κB p65 fluorescence intensity. (**d**) NF-κB p65 fluorescence intensity was quantified at day 50 (*n* = 11 for control group; *n* = 6 for IGU group). Scale bar: 100 µm. *p < 0.05. Error bars indicate SEM. An unpaired Student’s *t*-test was used for analyses.

As both COX-2 and NF-κB are critical for CNS inflammation, while iguratimod is considered a highly selective inhibitor for COX-2, we immunohistochemically examined COX-2 and NF-κB p65 in the chronic EAE spinal cord. Therapeutic iguratimod administration significantly decreased COX-2 and NF-κB p65 signal intensity in the spinal cord at the chronic phase of EAE compared with vehicle administration (*p* = 0.0277 for COX-2, *p* = 0.0375 for NF-κB, Fig. 4a–d). Taken together, these findings suggest that iguratimod initiated after the clinical onset can attenuate the disease severity of chronic EAE through the down-regulation of COX-2 and NF-κB p65 in CNS tissues.

### Iguratimod inhibits infiltration of Th1 and Th17 cells into the CNS and splenic Th1 and Th17 cell activation

**Figure 5:**
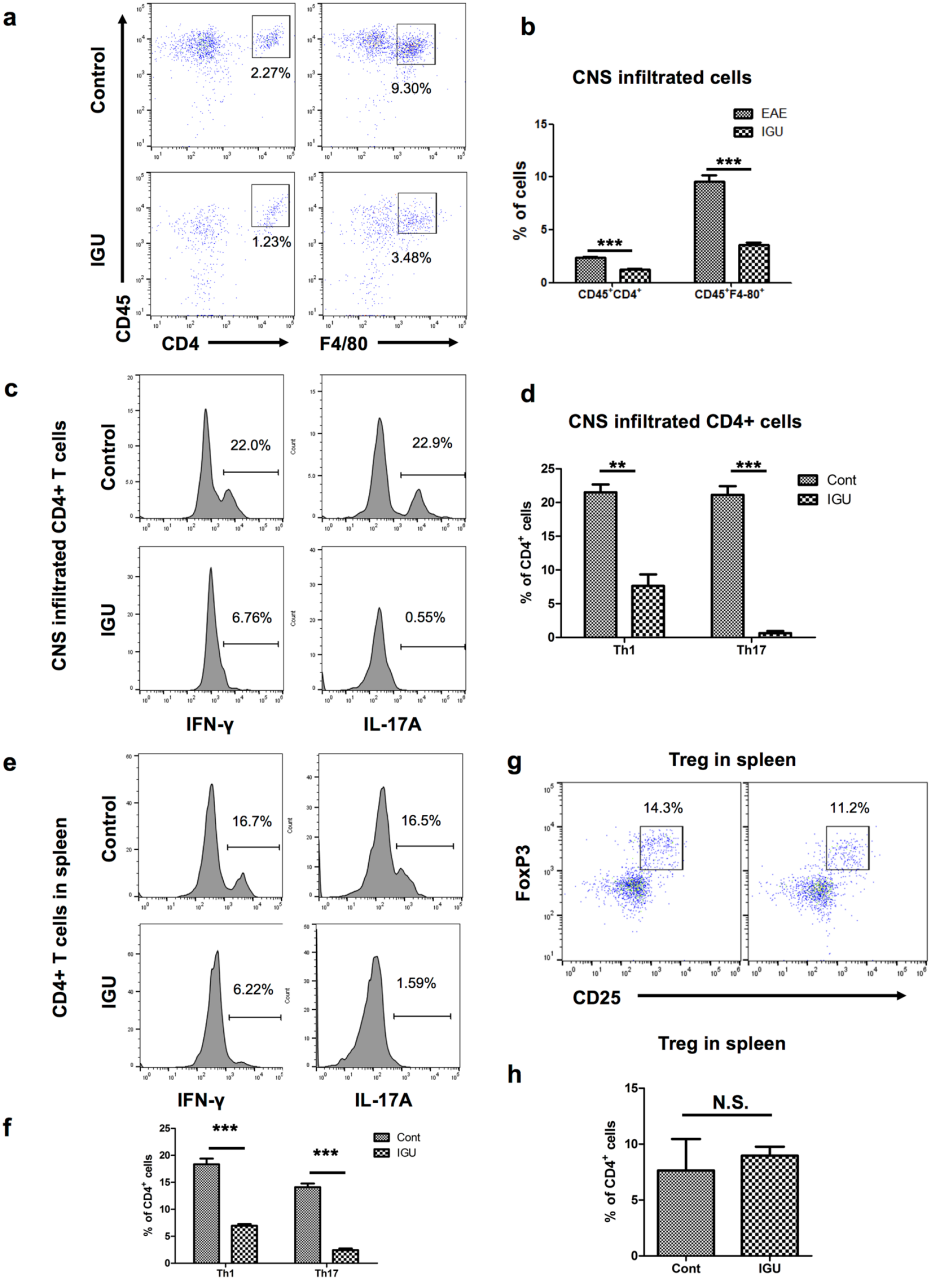
Flow cytometric analysis of macrophages and T cells in iguratimod-treated chronic EAE mice. (**a**) CD45^+^CD4^+^ T cells and CD45^+^F480^+^ macrophages in CNS tissues (brain and spinal cord) from one representative control or IGU-treated EAE mouse. Numbers on plots are percentages of double-positive cells among gated viable cells. Iguratimod decreases CD45^+^CD4^+^ T cells and CD45^+^F480^+^ macrophages in EAE CNS tissues. (**b**) Bar graphs showing percentages of CD45^+^CD4^+^ T cells and CD45^+^F480^+^ macrophages in CNS tissues (brain and spinal cord) from EAE mice. Data are summarised for 5 mice per group. (**c**) IFN-γ^+^CD4^+^ Th1 cells and IL-17A^+^CD4^+^ Th17 cells in CNS tissues (brain and spinal cord) from one representative control or IGU-treated EAE mouse. Numbers on plots are percentages of double-positive cells among MHC-II^−^CD4^+^ gated cells. Iguratimod decreases IFN-γ^+^CD4^+^ Th1 and IL-17A^+^CD4^+^ Th17 cell percentages among T cells infiltrating the EAE CNS. T cells were stimulated with PMA/ionomycin for 5 h. (**d**) Bar graphs showing percentages of IFN-γ^+^CD4^+^ Th1 cells and IL-17A^+^CD4^+^ Th17 cells among CD4^+^ T cells infiltrating the CNS tissues (brain and spinal cord) from EAE mice. Data are summarised for 5 mice per group. (**e**) IFN-γ^+^CD4^+^ Th1 cells and IL-17A^+^CD4^+^ Th17 cells in the spleen of one representative control or IGU-treated EAE mouse. Numbers on plots are percentages of double-positive cells among MHC-II^−^CD4^+^ gated cells. Iguratimod decreases IFN-γ^+^CD4^+^ Th1 and IL-17A^+^CD4^+^ Th17 cell percentages in the EAE spleen. T cells were stimulated with PMA/ionomycin for 5 h. (**f**) Bar graphs showing percentages of IFN-γ^+^CD4^+^ Th1 cells and IL-17A^+^CD4^+^ Th17 cells in the spleen of EAE mice. Data are summarised for 5 mice per group. (**g**) CD25^+^FoxP3^+^ Treg cells in the spleen of one representative control or IGU-treated EAE mouse. Numbers on plots are percentages of CD4^+^ gated cells. Iguratimod does not significantly change CD25^+^FoxP3^+^ Treg cell percentages in the EAE spleen. (**h**) Bar graphs showing percentages of CD25^+^FoxP3^+^ Treg cells in the spleen of EAE mice. Data are summarised for 5 mice per group. **p < 0.01, ***p < 0.0001, N.S. not significant. Error bars indicate SEM. An unpaired Student’s *t*-test was used for analyses.

To investigate the effects of iguratimod on T cell infiltration and splenic T cell activation, we measured interferon (IFN)-γ-producing Th1 cells, IL-17A-producing Th17 cells, and F4/80^+^ macrophages by flow cytometry among leukocytes isolated from CNS tissues of chronic EAE mice. Therapeutic iguratimod administration after the onset of EAE significantly decreased CD45^+^CD4^+^ T cell and CD45^+^F4/80^+^ macrophage infiltration into acute EAE CNS tissues compared with vehicle administration (*p* < 0.0001 for CD45^+^CD4^+^ T cells, *p* < 0.0001 for CD45^+^F4/80^+^ macrophages, Fig. 5a,b). Furthermore, iguratimod treatment decreased both IFN-γ^+^CD4^+^ Th1 and IL-17A^+^CD4^+^ Th17 cell percentages in CD4^+^ T cells among CNS-infiltrating T cells isolated from CNS tissues of acute EAE mice (*p* = 0.0002 for IFN-γ^+^CD4^+^ Th1 cells, *p* < 0.0001 for IL-17A^+^CD4^+^ Th17 cells, Fig. 5c,d).

We also measured splenic Th1, Th17, and FoxP3^+^ regulatory T (Treg) cells by flow cytometry. Iguratimod treatment significantly reduced Th1 and Th17 cells in the spleen compared with vehicle treatment (*p* < 0.0001 for IFN-γ^+^CD4^+^ Th1 cells, *p* < 0.0001 for IL-17A^+^CD4^+^ Th17 cells, Fig. 5e,f), but had no effect on CD25^+^FoxP3^+^ Treg cells (*p* = 0.6616, Fig. 5g,h). Taken together, these findings suggest that iguratimod initiated after the clinical onset can attenuate the disease severity of chronic EAE through inhibition of Th1 and Th17 cell infiltration into CNS tissues and splenic Th1 and Th17 cell activation.

### Iguratimod inhibits macrophage activation induced by LPS stimulation *in vitro*

**Figure 6:**
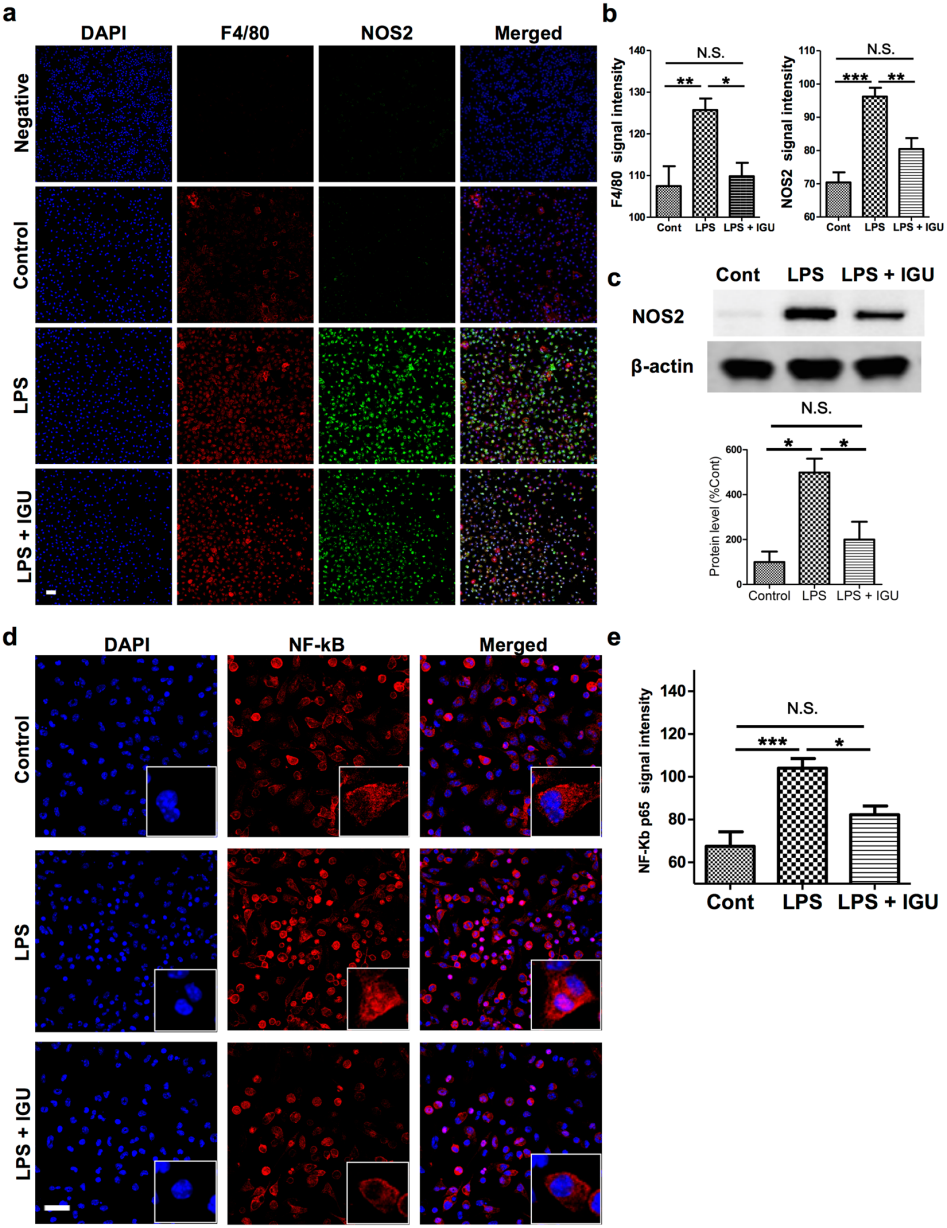
Iguratimod down-regulates the increased F4/80 and NOS2 expression in macrophages after LPS stimulation *in vitro*. (**a**) Representative images of F4/80 (red) and NOS2 (green) immunofluorescence in macrophages after stimulation with DMSO vehicle (control), LPS (10 ng/ml), or LPS (10 ng/ml) plus iguratimod (30 µg/ml) for 2 h. Cell nuclei were detected by DAPI (blue). Scale bar: 100 µm. (**b**) Quantification of F4/80 (*n* = 7 for control; *n* = 8 for LPS; *n* = 8 for LPS + IGU) and NOS2 (*n* = 11 for control; *n* = 11 for LPS; *n* = 10 for LPS + IGU) signal intensity in macrophages. (**c**) Protein levels of NOS2 in macrophages determined by western blotting after stimulation with DMSO vehicle (control), LPS (10 ng/ml), or LPS (10 ng/ml) plus iguratimod (30 µg/ml) for 24 h (*n* = 3 per group). Full length blots are presented in Supplementary Figure S2. (**d**) Representative images of NF-κB p65 immunofluorescence (red) in macrophages after stimulation with DMSO vehicle (control), LPS (10 ng/ml), or LPS (10 ng/ml) plus iguratimod (30 µg/ml) for 2 h. Cell nuclei were detected by DAPI (blue). Scale bar: 20 µm. (**e**) Quantification of NF-κB p65 signal intensity in macrophages (*n* = 6 for control; *n* = 5 for LPS; *n* = 6 for LPS + IGU). *p < 0.05, **p < 0.01, ***p < 0.0001. Error bars indicate SEM. One-way ANOVA with Tukey’s *post hoc* test was used for analyses.

To further elucidate the mechanisms underlying the amelioration of chronic EAE by iguratimod, we investigated the effects of iguratimod on activation of macrophages. Lipopolysaccharide (LPS), a major component of the outer membrane of Gram-negative bacteria, is one of the most powerful activators of macrophages, inducing the production of various pro-inflammatory mediators and even relapses in EAE^34^. We stimulated peritoneal macrophages for 2 h with 10 ng/ml LPS with or without 30 µg/ml iguratimod. As shown in Fig. 6a,b, control dimethyl sulfoxide (DMSO)-stimulated macrophages exhibited low levels of F4/80 immunofluorescence staining, a marker for macrophage activation. By contrast, LPS-stimulated macrophages without iguratimod treatment exhibited high levels of F4/80 immunofluorescence. However, a reduction in F4/80 staining was observed in LPS-stimulated macrophages with iguratimod treatment (Cont. vs. LPS, *p* < 0.01; LPS vs. LPS + IGU, *p* < 0.05; Fig. 6a,b). As pro-inflammatory macrophages produce high levels of nitric oxide (NO)^35^, we analysed the expression of NOS2 in LPS-stimulated macrophages. Similar to the case for F4/80, LPS-stimulated macrophages exhibited high levels of NOS2 immunofluorescence staining, and a reduction in NOS2 staining was observed in LPS-stimulated macrophages with iguratimod treatment (Cont. vs. LPS, *p* < 0.0001; LPS vs. LPS + IGU, *p* < 0.01; Fig. 6a,b). Inhibition of NOS2 expression by iguratimod treatment was confirmed by immunoblotting. Specifically, NOS2 protein levels were significantly increased in LPS-stimulated macrophages compared with control cells (*p* = 0.0067), while iguratimod treatment significantly reduced the NOS2 protein increase upon LPS stimulation (*p* = 0.0407, Fig. 6c). These findings indicate that iguratimod can inhibit the macrophage activation induced by LPS stimulation *in vitro*.

### Iguratimod inhibits NF-κB p65 nuclear translocation in LPS-stimulated macrophages *in vitro*

Since NF-κB plays a central role in the co-ordinated regulation of gene expression during macrophage activation^36^, we examined the effects of iguratimod on activation of the NF-κB signalling pathway. As activation of NF-κB is associated with nuclear translocation of the NF-κB p65 component^37^, we examined NF-κB p65 signal intensity in LPS-stimulated peritoneal macrophages by fluorescence confocal microscopy. NF-κB p65 was mainly localised in the perinuclear cytoplasm after control DMSO treatment. After 2 h of treatment with 10 ng/ml LPS, NF-κB p65 was significantly increased in LPS-stimulated macrophages compared with control cells (*p* < 0.0001), while iguratimod treatment significantly decreased NF-κB p65 upon LPS stimulation (*p* < 0.05, Fig. 6d,e). Taken together, these findings indicate that iguratimod suppresses macrophage activation, at least in part, through inhibition of NF-κB p65 nuclear translocation.

### Iguratimod inhibits microglial activation induced by LPS stimulation through an NF-κB pathway *in vitro*

**Figure 7:**
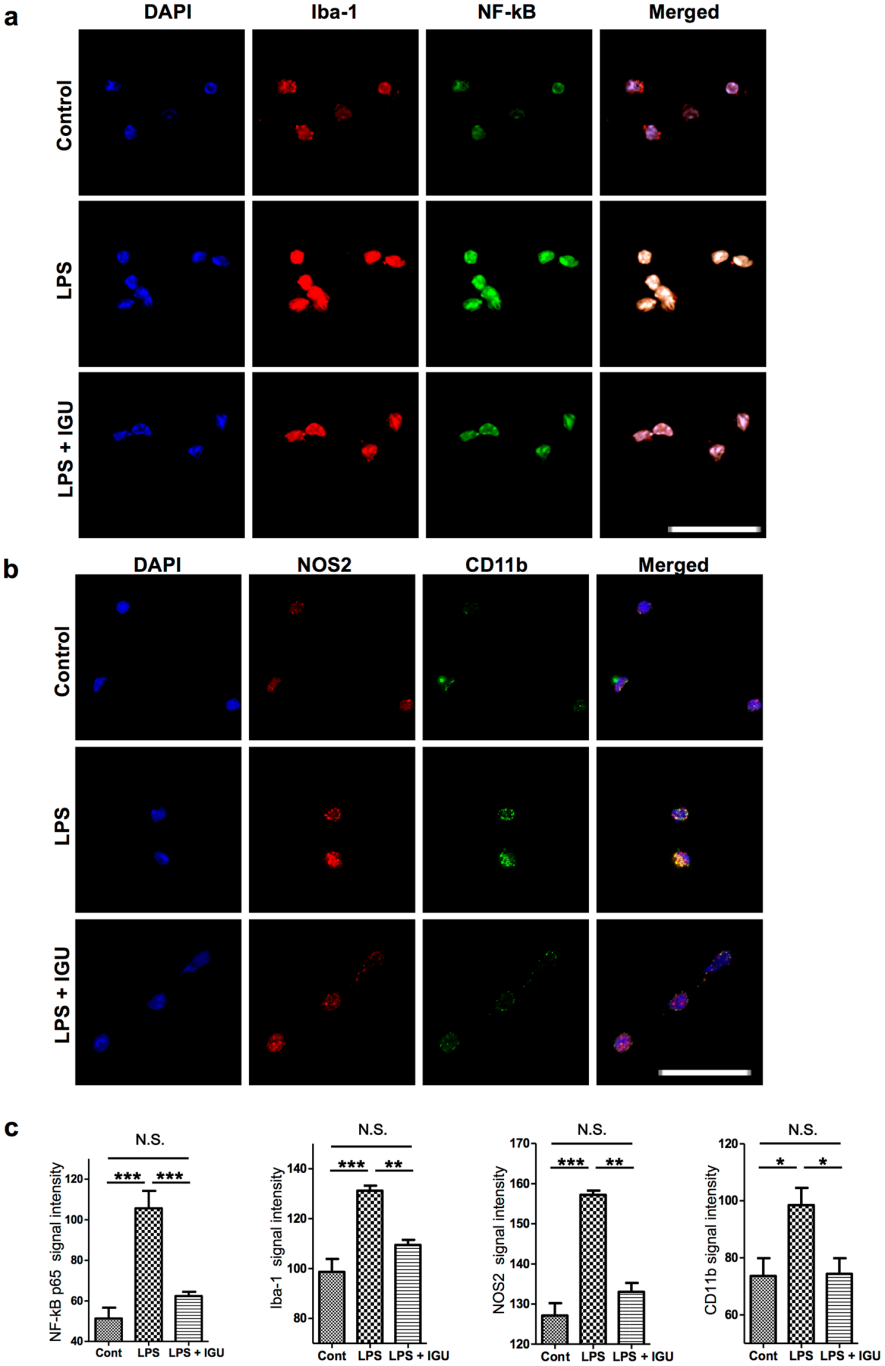
Iguratimod inhibits the up-regulation of NF-κB p65, Iba-1, NOS2, and CD11b in microglia after LPS stimulation *in vitro*. (**a**) Representative images of Iba-1 (red) and NF-κB p65 (green) immunofluorescence in microglia after stimulation with DMSO vehicle (control), LPS (10 ng/ml), or LPS (10 ng/ml) plus iguratimod (30 µg/ml) for 2 h. Cell nuclei were detected by DAPI (blue). Scale bar: 20 µm. (**b**) Representative images of NOS2 (red) and CD11b (green) immunofluorescence in microglia after stimulation with DMSO vehicle (control), LPS (10 ng/ml), or LPS (10 ng/ml) plus iguratimod (30 µg/ml) for 2 h. Cell nuclei were detected by DAPI (blue). Scale bar: 20 µm. (**c**) Quantification of NF-κB p65 (*n* = 5 for control; *n* = 6 for LPS; *n* = 7 for LPS + IGU), Iba-1 (*n* = 6 for control; *n* = 5 for LPS; *n* = 5 for LPS + IGU), NOS2 (*n* = 6 for control = 6, *n* = 6 for LPS; *n* = 4 for LPS + IGU), and CD11b (*n* = 6 for control; *n* = 5 for LPS; *n* = 5 for LPS + IGU) signal intensity in microglia. *p < 0.05, **p < 0.01, ***p < 0.0001. Error bars indicate SEM. One-way ANOVA with Tukey’s *post hoc* test was used for analyses.

We also investigated the effects of iguratimod on activation of microglia. LPS activates microglia as well as macrophages and induces pro-inflammatory mediators such as NO^38^. Similar to LPS-stimulated macrophages, LPS-stimulated microglia expressed higher levels of activation-associated cell surface markers, such as Iba-1 and CD11b, than control DMSO-stimulated microglia. Iguratimod treatment significantly reduced Iba-1 and CD11b fluorescence staining in LPS-stimulated microglia (Iba-1: Cont. vs. LPS, *p* < 0.0001; LPS vs. LPS + IGU, *p* < 0.01; CD11b: Cont. vs. LPS, *p* < 0.05; LPS vs. LPS + IGU, *p* < 0.05; Fig. 7a–c). NOS2 expression was also increased in LPS-stimulated microglia, and this up-regulation was reversed by iguratimod treatment (Cont. vs. LPS, *p* < 0.0001; LPS vs. LPS + IGU, *p* < 0.0001; Fig. 7b,c). Moreover, the up-regulation of NF-κB p65 by LPS was significantly inhibited by iguratimod treatment (Cont. vs. LPS, *p* < 0.0001; LPS vs. LPS + IGU, *p* < 0.05; Fig. 7a,c). These findings indicate that iguratimod inhibits activation of microglia as well as macrophages.

### Iguratimod inhibits up-regulation of M1 and some M2 genes induced by LPS stimulation in macrophages

**Figure 8:**
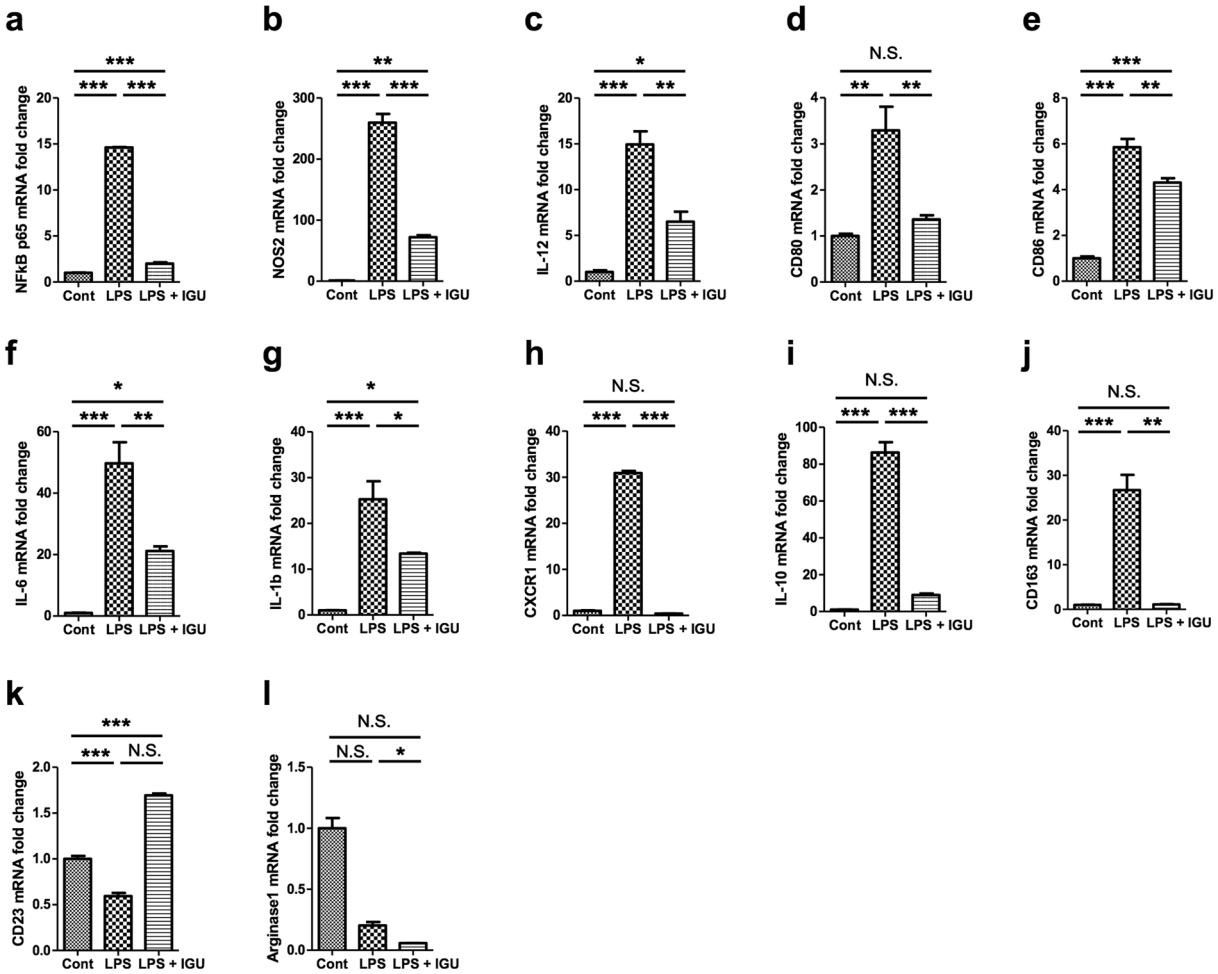
Phenotypes of activated macrophages (M1/M2) after stimulation with or without iguratimod *in vitro*. (**a**) NF-κB p65 transcripts were quantified by real-time PCR. (**b**–**g**) M1 marker gene transcripts were quantified by real-time PCR. (**b**), NOS2; (**c**), IL-12; (**d**), CD80; (**e**), CD86; (**f**), IL-6; (**g**), IL-1b. (**h**–**l**) M2 marker gene transcripts were quantified by real-time PCR. (**h**), CXCR1; (**i**), IL-10; (**j**), CD163; (**k**), CD23; (**l**), arginase-1; Values were normalised by GAPDH, and the fold changes compared with GAPDH are shown (*n* = 3 per group). *p < 0.05, **p < 0.01, ***p < 0.0001. Error bars indicate SEM. One-way ANOVA with Tukey’s *post hoc* test was used for analyses.

To characterise the anti-inflammatory effects of iguratimod on macrophage activation, we measured representative M1 and M2 gene expressions in macrophages by real-time PCR. First, we found that iguratimod significantly inhibited NF-κB expression induced by LPS in macrophages (Fig. 8a), consistent with the immunofluorescence staining results (Fig. 6d). Second, LPS markedly up-regulated M1 gene expressions such as NOS2, IL-12, CD80, CD86, IL-6, and IL-1β, and these increases were largely reversed by iguratimod treatment (Fig. 8b–g). Third, M2 gene responses against LPS varied from gene to gene. Some M2 genes, namely CXCR1, IL-10, and CD163, were up-regulated by LPS stimulation, and these increases were again reversed by iguratimod treatment (Fig. 8h–j). Regarding other M2 genes, CD23 was unchanged by LPS treatment, but significantly up-regulated by LPS + iguratimod treatment (Fig. 8k), while arginase-1 was significantly down-regulated by LPS treatment and unchanged by LPS + iguratimod treatment (Fig. 8l). Taken together, these findings indicate that iguratimod can strongly inhibit LPS-induced M1 gene up-regulation, while some M2 genes up-regulated by LPS stimulation are also down-modulated by iguratimod.

## Discussion

The main new findings of the present study are summarised as follows. First, iguratimod is highly efficacious for preventing not only acute, but also chronic EAE induced by MOG_35–55_ in C57BL/6 mice. Second, therapeutic administration of iguratimod after the onset of acute EAE can effectively improve chronic EAE clinically and histologically. Third, iguratimod can suppress LPS-induced pro-inflammatory activation of both macrophages and microglia by inhibiting nuclear translocation of NF-κB p65 *in vitro*. There are two previous studies on preventive administration of iguratimod for acute EAE^28,39^. Aikawa *et al*.^28^ reported that iguratimod suppressed the development of acute monophasic EAE induced by myelin basic protein (MBP) in Lewis rats until around day 30. They further found that iguratimod inhibited the production of IFN-γ, IL-6, and TNF-α by MBP-specific T cells after stimulation with MBP *in vitro*, suggesting that suppression of autoreactive T cells is responsible for the preventive effects of iguratimod in acute EAE. Bloom *et al*.^39^ examined the effects of iguratimod on the acute phase of EAE induced by MOG_35–55_ in C57BL/6 mice, and found that iguratimod combined with dexamethasone significantly ameliorated the clinical severity of acute EAE; however, they did not carry out histological examinations. The present findings expand these previous observations, and suggest the therapeutic potential of oral iguratimod for not only acute, but also chronic MS. In the present study, iguratimod was initiated after the clinical manifestations of EAE were confirmed in each animal, and indeed the EAE scores and body weight losses in the acute phase did not differ between the vehicle-treated and iguratimod-treated groups until day 13, with the EAE scores exceeding 2 in both groups. It is thus suggested that even iguratimod-treated mice had significant CNS damage caused by infiltrating inflammatory cells in the therapeutic regimen, and that it required at least 4 days for the therapeutic effects of iguratimod to become clinically apparent, i.e., around the peak of the disease and thereafter. Given that therapeutic drugs can be administered after appearance of symptoms, without waiting for the disease peak, in humans, we consider that our therapeutic regimen can be regarded as clinically relevant.

Initially, the mechanism for the anti-arthritis actions of iguratimod was considered to mainly involve COX-2 inhibition, resulting in reduced production of pro-inflammatory PGs, such as PGE2^40,41^. Indeed, we found a significant reduction of COX-2 in the chronic EAE spinal cord after therapeutic administration of iguratimod in the present study. We previously reported that PGE2 synthesis and PGE2 receptor (EP1, EP2, EP4) expression were elevated in acute MOG-EAE lesions^42^, and that both EAE mouse spinal cord and MS brain lesions exhibited dominant expression of microsomal PGE2 synthase 1 (mPGES-1) in macrophages^42^. Moreover, mPGES-1^−/−^ mice showed less severe EAE and lower production of IL-17 and IFN-γ than mPGES-1^+/+^ mice^42^. Therefore, the mPGES-1/PGE2/EP axis plays a critical role in acute EAE pathology, and blockade of COX-2 by iguratimod exerts beneficial effects on acute EAE and possibly on acute relapses in RRMS.

In chronic progressive MS, mounting evidence suggests that activated microglia and macrophages contribute to irreversible accumulation of neurological deficits. Specifically, diffuse injuries to normal-appearing white matter and cortical demyelination, hallmarks of progressive MS, are accompanied by profound microglial activation in the absence of florid perivascular cuffing^31,43^. Activated macrophages and microglia are also associated with persistent tissue disruption during the chronic phase of EAE^44,45^. In the present study, oral administration of iguratimod initiated after the disease onset significantly limited the disease progression, being correlated with decreases in demyelination, inflammatory cell infiltration, and microglial activation. These therapeutic effects of iguratimod lasted throughout the administration period. As MS relapses occur two to three times more frequently around common respiratory infections^46^, and LPS can directly induce EAE relapses via activation of CD4^−^ antigen-presenting cells with enhanced co-stimulatory molecule expression^34^, we investigated the effects of iguratimod on LPS-induced activation of macrophages/microglia. We found that not only peritoneal macrophages, but also isolated microglia were suppressed by iguratimod after LPS application, as shown by the down-modulation of F4/80, NOS2, or Iba-1, in association with inhibition of nuclear translocation of NF-κB p65. Our findings are in accord with the suppression of an NF-κB pathway by iguratimod in a human monocytic leukaemia cell line (THP-1)^26,27^ and cultured human synovial cells^47,48^, and in line with the identification of iguratimod as an inhibitor of macrophage migration inhibitory factor^39^. The crucial role of NF-κB in neuroinflammation is supported by the observation that EAE is markedly attenuated in NF-κB-deficient mice^49^. As the concentration of iguratimod in the brain and spinal cord reaches about 10% of the peripheral blood levels after oral administration^50^, direct NF-κB blockade by iguratimod in microglia as well as macrophages may play an important role in the treatment of chronic EAE. Indeed, therapeutic administration of iguratimod significantly decreased NF-κB p65 in the chronic EAE spinal cord in the present study. In addition, NO, metabolically produced by NOS, has direct neurotoxic effects that have been linked to tissue damage in chronic EAE^43,51^. Suppression of NF-κB-induced NOS2 production by iguratimod surely contributes to the amelioration of chronic EAE.

In addition to its effects on macrophages/microglia, iguratimod may act directly on inflammatory cells in both the periphery and the CNS, even in chronic EAE, because iguratimod was found to inhibit proliferation of autoreactive CD3^+^ T cells and production of pro-inflammatory cytokines from peripheral immune cells in rat acute monophasic EAE and human rheumatoid arthritis^28,34^. In the present study, flow cytometric analyses of inflammatory cells isolated from chronic EAE CNS tissues revealed decreases in not only CD45^+^F4/80^+^ macrophages, but also IFN-γ^+^CD4^+^ Th1 cells and IL-17A^+^CD4^+^ Th17 cells after iguratimod treatment. Moreover, splenic Th1 and Th17 cells, but not Treg cells, were suppressed by iguratimod in the chronic phase of EAE. Taken together, these findings suggest that iguratimod initiated after the clinical onset can attenuate the disease severity of chronic EAE, partly through inhibition of Th1 and Th17 cell infiltration into CNS tissues and splenic Th1 and Th17 cell activation. Therefore, we consider that inhibition of both macrophages/microglia and CD3^+^ T cells is crucial for alleviation of EAE. However, in chronic EAE, CD3^+^ T cells were markedly decreased compared with acute EAE (roughly 25% of the level in acute EAE), while Iba-1^+^ cells corresponded to 80% of the level in acute EAE on average, suggesting that suppression of macrophages/microglia in the CNS by iguratimod through inhibition of an NF-κB pathway becomes increasingly important in the chronic phase.

Recent DMDs targeting the adaptive immune system, such as fingolimod, dimethyl fumarate, natalizumab, and anti-CD20 monoclonal antibodies, are highly effective for RRMS, but occasionally cause the fatal complication PML^52–56^. Iguratimod has been widely used for rheumatoid arthritis without occurrence of PML or severe side effects^23–25^. As our study revealed beneficial effects of therapeutic iguratimod administration on acute and chronic EAE, particularly in the chronic phase, a clinical trial of iguratimod in RRMS and SPMS, the latter of which has few effective therapies, is warranted in the future.

## Methods

### Mice and ethical statement

C57BL/6 female mice were purchased from KBT Oriental (Tosu, Japan). All mice were bred and maintained under specific pathogen-free conditions in the Center of Biological Research, Graduate School of Medical Sciences, Kyushu University. All experiments were carried out according to the guidelines for proper conduct of animal experiments published by the Science Council of Japan, as well as the ARRIVE (Animal Research: Reporting of *In Vivo* Experiments) guidelines for animal research. Ethical approval for the study was granted by the Animal Care and Use Committee of Kyushu University (#A26–042).

### Induction and clinical evaluation of EAE

EAE was induced in 11-week-old female C57BL/6 J mice as described^57^. Briefly, C57BL/6 J mice received a subcutaneous injection of 200 µg MOG_35–55_ peptide in 50 µl of PBS emulsified in an equal volume of CFA containing 1 mg/ml *Mycobacterium tuberculosis* H37RA (#231131; BD Difco, Lawrence, KS) on day 0, and intraperitoneal injections of 200 ng pertussis toxin (#180-A1; List Biological Laboratories Inc., Campbell, CA) in 0.2 ml of PBS on day 0 and day 2. Mice were examined daily for signs of EAE and scored as follows: 0, no signs; 1, limp tail; 2, hind limb weakness; 3, hind limb paralysis; 4, hind limb plus forelimb paralysis; 5, moribund.

### Iguratimod treatment *in vivo*

Iguratimod (Toyama Chemical Co. Ltd., Tokyo, Japan) was suspended in 100 µl of 0.5% (w/v) CMC solution and orally administered once-daily (30 mg/kg/day) from day 0 to day 50 for preventive treatment experiments or twice-daily (50 mg/kg/day) from day 10 to day 50 for therapeutic treatment experiments. The same amounts of CMC solution were administered to control mice.

### Histological and immunohistochemical analyses

Mice were anesthetised by isoflurane (Pfizer Japan Inc., Tokyo, Japan), and perfused transcardially with PBS followed by ice-cold 4% paraformaldehyde. The spinal cord was removed and processed by sequential placement in 15% sucrose/PBS and 30% sucrose/PBS for 24 h at 4 °C. After the samples were frozen in criopreservation buffer (OCT compound, Sakura fine tec, Tokyo, Japan) at −25 °C, transverse spinal cord sections (20 µm) were cut from on a Leica CM 1850 cryostat (Leica Microsystems Gmbh, Wetzlar, Germany), incubated for 2 h at room temperature in a blocking solution (0.1% Triton X-100 in PBS [PBS-T] with 10% normal goat serum), and incubated at 4 °C with primary antibodies for 12 h. Detailed information for the primary antibodies is provided in Table S1. Next, the sections were incubated with secondary antibodies conjugated to Alexa Fluor 488 or 594 (1:1000; Thermo Fisher, Rockford, IL) and 4′,6-diamidino-2-phenylindole (DAPI) (Sigma-Aldrich, Tokyo, Japan), and mounted with Permafluor (#TA-030-FM; Thermo Scientific, Fremont, CA). Images were captured using a confocal laser microscope system (Nikon A1; Nikon, Tokyo, Japan).

### Quantification of myelin density and cell infiltration in the spinal cord

The images for myelin staining were converted to grayscale and analysed by density measurement with ImageJ version 1.6.0_24 (Windows version of NIH Image; downloaded from https://imagej.nih.gov/ij/download.html). Infiltrated cells in the spinal cord were measured by the area ratio of positive cells to the whole spinal cord. The results for each experimental condition were averaged from five unilateral levels per mouse.

### Microglial circularity analysis

The circularity of microglia was calculated automatically by ImageJ (circularity = 4πS/L2). Cells with circularity close to 1 were regarded to have a morphology close to round cells, indicating an activated state^58^.

### Flow cytometric analysis

EAE mice were treated with or without iguratimod once-daily (30 mg/kg/day) from day 0 to day 15. At day 15, mice were euthanised and transcardially perfused with 30 ml of PBS, and their brains and spinal cords were harvested. Single-cell suspensions were prepared as described^57^. Briefly, mononuclear cells (MNCs) were harvested by Percoll (Sigma-Aldrich, St. Louis, MO) gradient (70%/30%) centrifugation at 500 × *g* and 18 °C for 40 min and suspended in RPMI complete medium (#189-02025; Wako, Osaka, Japan; supplemented with 10% heat-inactivated foetal calf serum, 100 U/ml penicillin, and 100 µg/ml streptomycin). Viable cells were counted after staining with 0.4% Trypan blue (>95% of cells were alive), as described^57^. For surface-marker staining, cells were incubated with fluorochrome-conjugated antibodies against CD4, CD45, and F4/80 for 30 min on ice, and analysed by flow cytometry using a Sony SH-800 (Sony Corporation, Tokyo, Japan). Splenocytes were also harvested as described^59^. To analyse Th1 and Th17 cells, splenocytes or CNS-infiltrating MNCs were stimulated with 10 ng/ml PMA and 1 µg/ml ionomycin (in 200 μl of RPMI medium) in 96-well dishes for 5 h, and then surface-stained with a monoclonal antibody against CD4. After fixation and permeabilisation with Fix & Perm Medium (Invitrogen, Frederick, MD), intracellular cytokines were stained with antibodies against IL-17A or IFN-γ (BD Biosciences, San Jose, CA). For Treg cell analysis, FoxP3 staining was carried out using an anti-Mouse/Rat Foxp3 Staining Set FITC (eBioscience, San Diego, CA), in accordance with manufacturer’s instructions. Briefly, splenocytes were incubated with antibodies against CD4 and CD25 for 30 min at 4 °C, fixed with Fixation/Permeabilization working solution for 30 min, washed with 1× Permeabilization Buffer, and incubated with an anti-FoxP3 monoclonal antibody for 30 min. The stained cells were subjected to flow cytometric analysis using the Sony SH-800.

### Peritoneal macrophage isolation, culture, and treatment

Ten-week-old C57BL/6 J female mice were euthanised immediately prior to the procedure, and 5 ml of sterile PBS was injected into the peritoneal cavity using a 25-gauge needle. The entire body was gently shaken for 10 s, and the PBS containing resident peritoneal cells was withdrawn using a 23-gauge needle. The cells were centrifuged at 1,000 rpm and 4 °C for 5 min, washed with complete RPMI medium, and seeded into 10-cm dishes or collagen type I cellware 8-well culture slides (#354603; BioCoat, Bedford, MA) at a density of 2 × 10^6^ cells/ml. The dishes or culture slides were placed in an incubator at 37 °C under 5% CO_2_ for 45 min to allow macrophage adherence. The medium was exchanged to remove non-attached cells. The macrophages were treated with DMSO vehicle (control), 10 ng/ml LPS in DMSO, or 10 ng/ml LPS plus 30 µg/ml iguratimod in DMSO for 2 h for immunofluorescence examination or 24 h for immunoblotting analysis.

### Microglia isolation, culture, and treatment

The MNCs isolated by Percoll gradient (70%/30%) centrifugation were stained with anti-F4/80-phycoerythrin and anti-CD45-PerCP antibodies, and sorted with the Sony SH-800 by gating on F4/80^+^CD45^dim^ as microglia. The sorted microglia were cultured in collagen type I cellware 8-well culture slides at a density of 10,000 cells/ml in RPMI complete medium. The microglia were treated with DMSO vehicle (control), 10 ng/ml LPS in DMSO, or 10 ng/ml LPS plus 30 µg/ml iguratimod in DMSO for 2 h.

### Immunofluorescence staining and fluorescence intensity measurement

Macrophages or microglia were fixed with 4% (w/v) paraformaldehyde/PBS for 20 min at room temperature, washed three times with PBS, permeabilised with PBS-T for 15 min at room temperature, washed three times with PBS, and blocked with PBS-T plus 10% goat serum for 1 h at room temperature. The primary antibodies were directly added to the blocking solution, and the slides were gently shaken overnight at 4 °C. The cells were washed three times with PBS, and incubated with secondary antibodies and DAPI overnight at 4 °C. Finally, the cells were washed and mounted with Permafluor. Images were captured using the Nikon A1 confocal laser microscope system. The images were converted to grayscale and analysed by intensity measurement with ImageJ.

### Immunoblotting

Peritoneal macrophages were cultured at a density of 2 × 10^6^ cells/ml in 10-cm dishes, and treated with DMSO vehicle (control), 10 ng/ml LPS in DMSO, or 10 ng/ml LPS plus 30 µg/ml iguratimod in DMSO for 24 h. The cells were washed with PBS, collected into 1.5-ml tubes, snap-frozen in liquid nitrogen, and stored at −80 °C until analysis. Proteins were extracted from these peritoneal macrophages as described^60^. Briefly, cells were homogenised in RIPA buffer by sonication at 20 kHz and centrifuged at 10,000 × *g* for 10 min at 4 °C. The protein concentrations were determined using a Pierce™ BCA Protein Assay Kit (Thermo Fisher Scientific, Rockford, IL). The proteins were electrophoresed in 7.5% mini PROTEAN precast gels (#4561026; Bio-Rad, Hercules, CA), transferred onto PVDF membranes, and incubated at 4 °C overnight with a primary antibody against NOS2. After washing, the membranes were incubated with 0.5% horseradish peroxidase-labelled IgG. The membrane-bound antibodies were detected with an enhanced chemiluminescence detection kit (ECL™ Prime Western Blotting System; RPN2232; GE Healthcare, Buckinghamshire, UK) and analysed with an image analyser (ChemiDoc XRS System; Bio-Rad).

### RNA isolation and gene expression analysis

Total RNA was extracted from isolated macrophages with an RNeasy Mini Kit (#74104; Qiagen, Hilden, Germany). RNA was reverse-transcribed by ReverTra Ace® qPCR Master Mix with gDNA remover (#FSQ-301; Toyobo, Osaka, Japan) and used for quantitative analysis with an ABI PRISM 7500 TaqMan apparatus (Applied Biosystems, Foster City, CA). All values were normalised by expression of GAPDH. The primer sets are listed in Table S2.

### Statistics

All data are presented as mean ± SEM. The statistical significance of differences between values was determined by an unpaired *t*-test, Mann–Whitney U test, or one-way ANOVA. Values of *p* < 0.05 were considered statistically significant.

## Electronic supplementary material

Supplementary material
