# Supplementary material for: Novel disease-modifying anti-rheumatic drug iguratimod suppresses chronic experimental autoimmune encephalomyelitis by down-regulating activation of macrophages/microglia through an NF-κB pathway
Source: Sci Rep. 2018 Jan 31;8:1933. doi: 10.1038/s41598-018-20390-5 (PMC5792543; doi:10.1038/s41598-018-20390-5)
Supplement: Supplementary file 1 — Supplementary material [file 41598_2018_20390_MOESM1_ESM.pdf]

Supplementary information

Novel disease-modifying anti-rheumatic drug iguratimod suppresses chronic experimental autoimmune encephalomyelitis by down-regulating activation of macrophages/microglia through an NF-kB pathway

Abbreviated title: Iguratimod ameliorates chronic experimental autoimmune encephalomyelitis

Guangrui Li[1], Ryo Yamasaki[1]*, Mei Fang[1], Katsuhisa Masaki[1], Hirofumi Ochi[2], Takuya Matsushita1, Jun-ichi Kira[1]*

[1]Department of Neurology, Neurological Institute, Graduate School of Medical Sciences, Kyushu University, Fukuoka 812-8582, Japan
[2]Department of Geriatric Medicine and Neurology, Ehime University Graduate School of Medicine, Matsuyama 791-0295, Japan

*Co-corresponding authors: Ryo Yamasaki and Jun-ichi Kira, Department of Neurology, Neurological Institute, Graduate School of Medical Sciences, Kyushu University, 3-1-1 Maidashi, Higashi-ku, Fukuoka 812-8582, Japan.
Phone: +81-92-642-5340
Fax: +81-92-642-5352
E-mail: kira@neuro.med.kyushu-u.ac.jp.

Figure S1

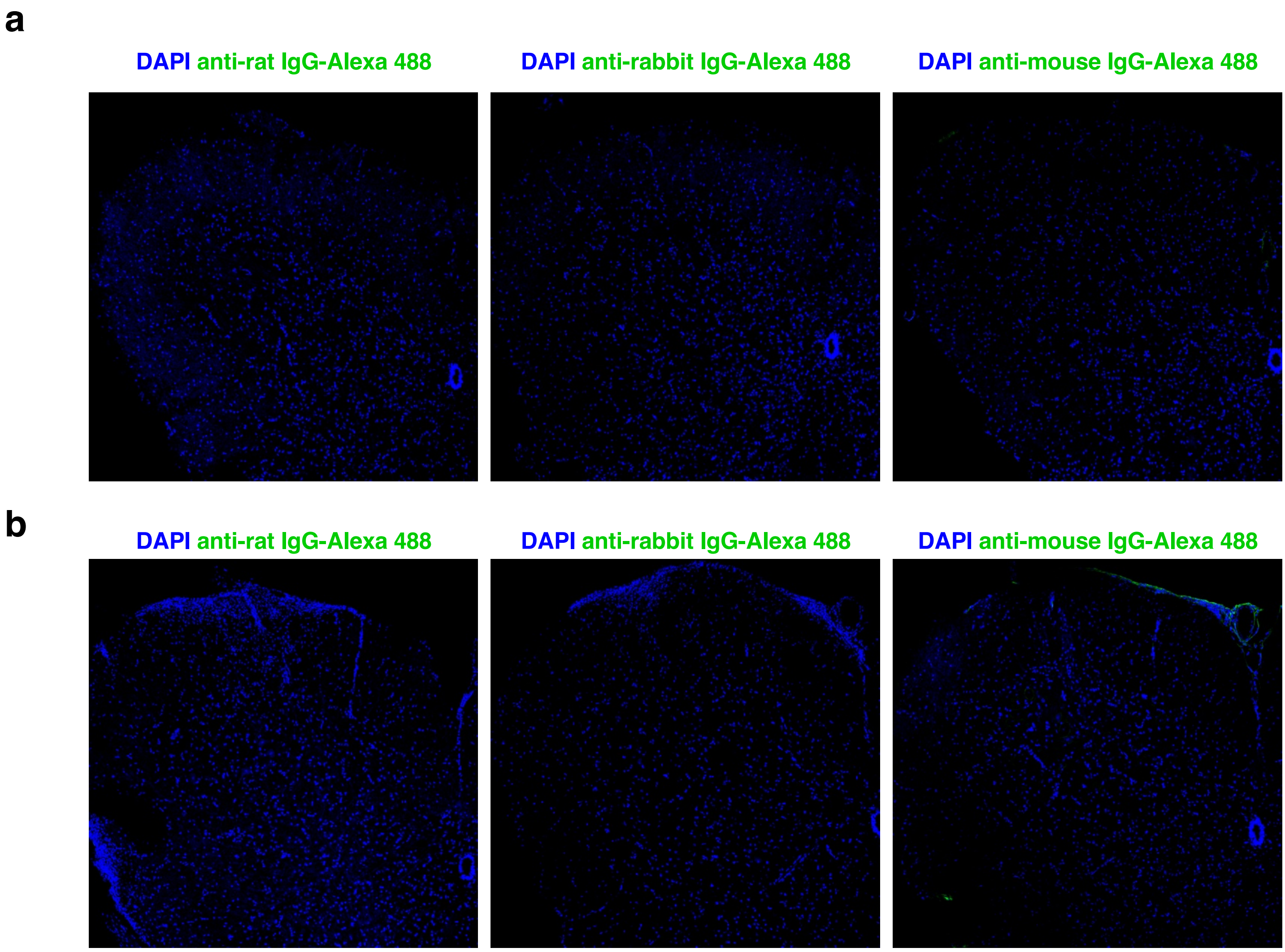

Figure S1. Negative control images for immunofluorescence staining. (**a**) Spinal cord samples from EAE mice with preventive iguratimod treatment (day 15) stained with anti-rat IgG-Alexa 488, anti-rabbit IgG-Alexa 488, and anti-mouse IgG-Alexa 488 antibodies. (**b**) Spinal cord samples from EAE mice with therapeutic iguratimod treatment (day 50) stained with anti-rat IgG-Alexa 488, anti-rabbit IgG-Alexa 488, and anti-mouse IgG-Alexa 488 antibodies.

Figure S2

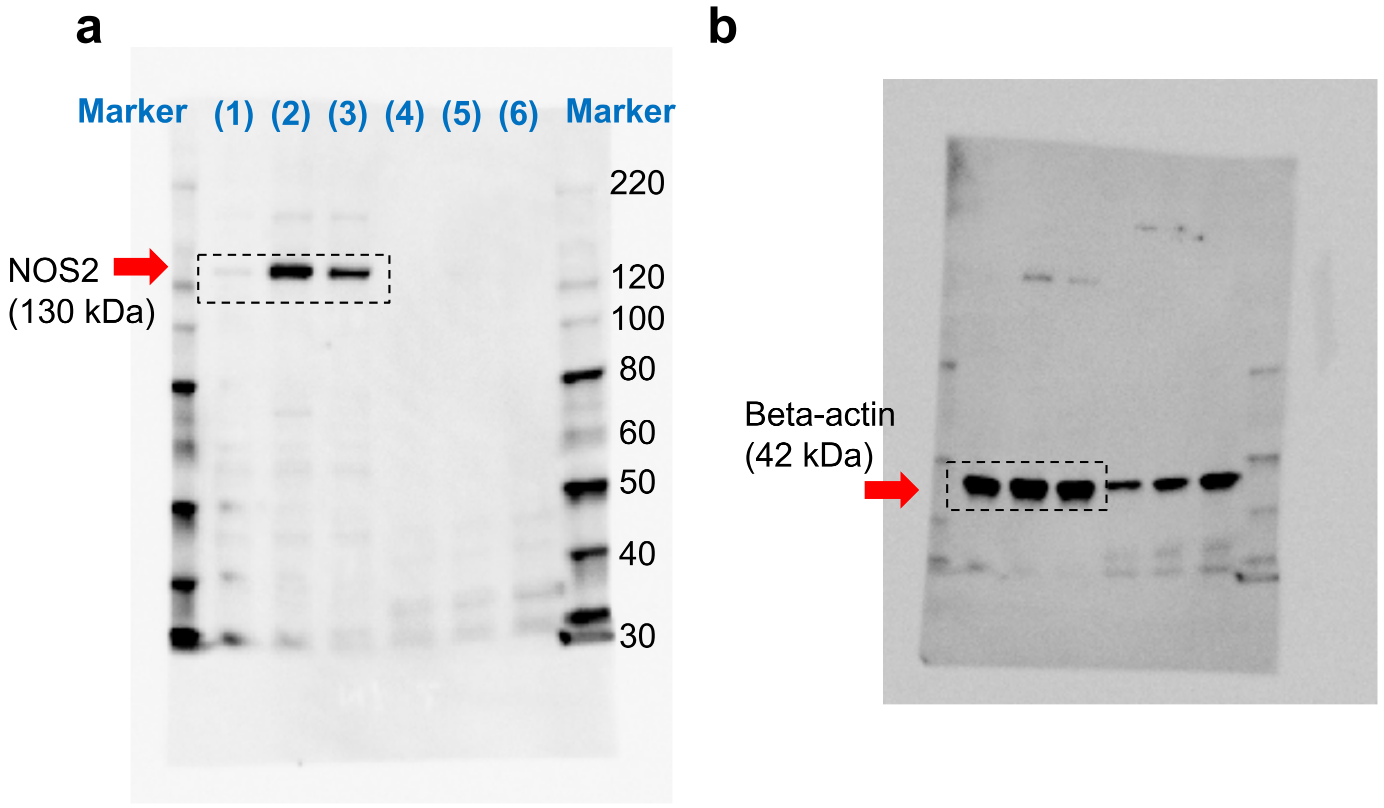

Figure S2. Full-length blots image of NOS2 western blot from peritoneal macrophages and splenocytes (Figure 6c).

5×10^6 cells in each 6 cm dish were cultured for 24 h, at the concentration of 1×10^6 /ml. Cells were then washed with ice–cold PBS, and harvested into a 1.5 ml tube. Cells were lysed with RIPA buffer. Electrophoresis was performed by a bio-rad western blot system. 7.5% gel was used. After electrophoresis, semi-dry method was used for transferring protein from gel to PVDF membrane. The membrane was first stained by anti-NOS2 antibody **(a)** and anti-beta-actin antibody after antibody stripping **(b)**. Dotted lines indicate areas used in main figure 6c. Numbers on the top of each lane indicates sample characteristics; (1, 2, 3): peritoneal macrophage, (4, 5, 6): splenocytes. (1, 4): negative control, (2, 5): 10 ng/ml LPS, (3, 6): 10 ng/ml LPS + 30 μg/ml Iguratimod. Results of quantification analysis was showed in Figure 6c.

Table S1. Antibodies used in this study

Antigen	Clone	Type	Dilution	Buffer	Incubation condition	Time	Source
Arginase	43-81(m)	Mouse IgG1, κ	1:1,000	10% BSA in TBS-0.1% Tween-20	4°C	overnight	Santa Cruz Biotechnology
CD11b	M1/70	Rat IgG2b, κ	1:200	10% BSA in TBS-0.1% Tween-20	4°C	overnight	BD Pharmingen
CD169	MOMA-1	Rat IgG2a	1:200	10% BSA in TBS-0.1% Tween-20	4°C	overnight	Bio-Rad
CD3 molecular complex	17A2	Rat IgG2b	1:1,000	10% BSA in TBS-0.1% Tween-20	4°C	overnight	BD Pharmingen
CD45	IBL3/16	Rat IgG1	1:1,000	10% BSA in TBS-0.1% Tween-20	4°C	overnight	Bio-Rad AbD Serotec
COX-2	H-3	Mouse IgM, κ	1:200	10% BSA in TBS-0.1% Tween-20	4°C	overnight	Santa Cruz Biotechnology
F4/80	CI:A3-1	Rat IgG2b	1:1,000	10% BSA in TBS-0.1% Tween-20	4°C	overnight	Bio-Rad AbD Serotec
Fluoromyelin red	-	-	1:100	10% BSA in TBS-0.1% Tween-20	Room temperature	4 hours	Thermo Fisher Scientific
Iba-1	Polyclonal	Rabbit IgG	1:1,000	10% BSA in TBS-0.1% Tween-20	4°C	overnight	Wako
NF-kB p65	Polyclonal	Rabbit	1:1,000	10% BSA in TBS-0.1% Tween-20	4°C	overnight	Santa Cruz Biotechnology
NOS2	Polyclonal	Rabbit	1:1,000	10% BSA in TBS-0.1% Tween-20	4°C	overnight	Santa Cruz Biotechnology
APC-conjugated anti-CD25	PC61.5	Rat IgG1λ	1:100	1% BSA and 0.1% sodium azide in PBS	4°C	30 min	eBioscience
FITC-conjugated anti-CD4	RM4-5	Rat IgG2a, κ	1:100	1% BSA and 0.1% sodium azide in PBS	4°C	30 min	Sony
FITC-conjugated anti-FoxP3	FJK-16s	Rat IgG2a, κ	1:100	1% BSA and 0.1% sodium azide in PBS	4°C	30 min	eBioscience
FITC-conjugated anti-IFN-γ	XMG1.2	Rat IgG1, κ	1:100	1% BSA and 0.1% sodium azide in PBS	4°C	30 min	eBioscience
PerCP-conjugated anti-CD45	OX-1	Mouse IgG1, κ	1:100	1% BSA and 0.1% sodium azide in PBS	4°C	30 min	Biolegend
PE-conjugated anti-CD4	GK1.5	Rat IgG2b, κ	1:100	1% BSA and 0.1% sodium azide in PBS	4°C	30 min	BD Pharmingen
PE-conjugated anti-F4/80	BM8	Rat IgG2a, κ	1:100	1% BSA and 0.1% sodium azide in PBS	4°C	30 min	Biolegend
PE/Cy7-conjugated anti-IL-17a	TC11-18H10.1	Rat IgG1, κ	1:100	1% BSA and 0.1% sodium azide in PBS	4°C	30 min	Sony
Beta-Actin	AC-15	Mouse IgG1, κ	1:1000	1% BSA and 0.1% sodium azide in PBS	4°C	30 min	SIGMA-ALDRICH

Table S2. Primers used for qPCR in this study

	Forward (5'–3')	Reverse (5'–3')
NF-kB p65	AGGCTTCTGGGCCTTATGTG	TGCTTCTCTCGCCAGGAATAC
NOS2	GTTCTCAGCCCAACAATACAAGA	GTGGACGGGTCGATGTCAC
IL-12a	CAATCACGCTACCTCCTCTTTT	CAGCAGTGCAGGAATAATGTTTC
IL-6	CTGCAAGAGACTTCCATCCAG	AGTGGTATAGACAGGTCTGTTGG
IL-1b	GAAATGCCACCTTTTGACAGTG	TGGATGCTCTCATCAGGACAG
CD80	GCTGTGTCGTTCAAAAGAAGGA	TGGGAAATTGTCGTATTGATGCC
CD86	CTGGACTCTACGACTTCACAATG	AGTTGGCGATCACTGACAGTT
IL-10	AGCCTTATCGGAAATGATCCAGT	GGCCTTGTAGACACCTTGGT
IL-2Ra	AACCATAGTACCCAGTTGTCGG	TCCTAAGCAACGCATATAGACCA
CXCR1	CCCCTGTGCAAGATGGTCTC	GGCGGAAGATAGCAAAAGGCA
Arginase-1	CTCCAAGCCAAAGTCCTTAGAG	AGGAGCTGTCATTAGGGACATC
CD23	ATCTCAGCCGTGATCTTGTTCT	ACCATACAAAAACAGGACAGCAT
CD163	TGTGCAGTAACGGCTGGAG	ATCATGTTTGCAGTCCCAAAGA
GAPDH	TGGCCTTCCGTGTTCCTAC	GAGTTGCTGTTGAAGTCGCA
